# The Millennia-Long Development of Drugs Associated with the 80-Year-Old Artificial Intelligence Story: The Therapeutic Big Bang?

**DOI:** 10.3390/molecules29122716

**Published:** 2024-06-07

**Authors:** Aurore Crouzet, Nicolas Lopez, Benjamin Riss Yaw, Yves Lepelletier, Luc Demange

**Affiliations:** 1 UMR 8038 CNRS CiTCoM, Team PNAS, Faculté de Pharmacie, Université Paris Cité, 4 Avenue de l’Observatoire, 75006 Paris, France; 2 W-MedPhys, 128 Rue la Boétie, 75008 Paris, France; 3 ENOES, 62 Rue de Miromesnil, 75008 Paris, France; 4 Unité Mixte de Recherche «Institut de Physique Théorique (IPhT)» CEA-CNRS, UMR 3681, Bat 774, Route de l’Orme des Merisiers, 91191 St Aubin-Gif-sur-Yvette, France; 5 Université Paris Cité, Imagine Institute, 24 Boulevard Montparnasse, 75015 Paris, France; 6 INSERM UMR 1163, Laboratory of Cellular and Molecular Basis of Normal Hematopoiesis and Hematological Disorders: Therapeutical Implications, 24 Boulevard Montparnasse, 75015 Paris, France

**Keywords:** artificial intelligence, drug discovery, drug development, drug design

## Abstract

The journey of drug discovery (DD) has evolved from ancient practices to modern technology-driven approaches, with Artificial Intelligence (AI) emerging as a pivotal force in streamlining and accelerating the process. Despite the vital importance of DD, it faces challenges such as high costs and lengthy timelines. This review examines the historical progression and current market of DD alongside the development and integration of AI technologies. We analyse the challenges encountered in applying AI to DD, focusing on drug design and protein–protein interactions. The discussion is enriched by presenting models that put forward the application of AI in DD. Three case studies are highlighted to demonstrate the successful application of AI in DD, including the discovery of a novel class of antibiotics and a small-molecule inhibitor that has progressed to phase II clinical trials. These cases underscore the potential of AI to identify new drug candidates and optimise the development process. The convergence of DD and AI embodies a transformative shift in the field, offering a path to overcome traditional obstacles. By leveraging AI, the future of DD promises enhanced efficiency and novel breakthroughs, heralding a new era of medical innovation even though there is still a long way to go.

## 1. Introduction

Drug discovery (DD) is a very costly and time-consuming process that can cost up to USD 4.54 billion and can take up to 12 years to bring a drug to market. Another issue is that the development of drugs is subject to a lot of failures. Therefore, DD can be a high-risk and high-stakes venture [1]. Artificial Intelligence (AI) is a field that is inspired by how the human brain functions. The first research into what we know as AI dates from the 20th century. With the possibility to explore the models of AI due to the power of current machines, AI is now at the centre of attention with prominent uses in speech recognition, image recognition, and movie and text generation. The emergence of ChatGPT software has created an important stimulation of AI [2,3] that is now a concern worldwide. AI is used as a tool in many companies from banks to biotechnology companies [4,5,6]. The use of AI in DD is promising, knowing that there is a number between 10^23^ and 10^66^ of drug-like components [7] to explore. The size of this search space almost mandates AI as it has proven useful with many Big Data issues. 

This review article focuses on AI and DD; its first goal is to put forward the history of these fields to have some understanding of their evolutions. This review is more specifically on the use of AI to help discover new drug candidates (DCs) and it will help in understanding the challenges surrounding the use of AI for DD. The discussion is divided into different sections that gradually leads to the full understanding of the opportunity that is AI for DD. First of all, we focus on DD and drug development by explaining DD’s history, modern DD, and the impact of the arrival of AI. Then, we focus on AI from history, its use, and finally the explanation of relevant models within the context of DD. The third part zooms in on the use of AI for DD, describing its challenges and detailing some uses in medicine, and it provides a quick overview of databases used in this process. Eventually, we have a look at some recent uses of AI for DD by putting forward some articles that successfully used AI to find interesting DCs or structures that are promising. We focus on articles that went from *in silico* to wet-lab experiment in order to shed light on works that are both theoretically and experimentally viable (or validated). 

This review is not meant to be an exhaustive listing of all methods used in AI or a methodology to develop an AI tool for DD, since others have recently published articles on this subject [8,9,10]. However, our ambition is to create a consistent review on AI and DD by going from the sources of the creation to today’s practices, hoping to encourage the use of AI to discover new drugs.

## 2. Drug Discovery and Development

### 2.1. History and Market

To better understand how DD has evolved through time and why the arrival of new technologies has been such a revolution, a point on its related history and market is necessary. It is important to point out the human process of DD. When trying to develop a drug for a specific disease, the challenges of DD are numerous. These include the understanding of the causes for the disease and/or the underlying processes that lead the patient to develop a disease. Moreover, this may lead to the discovery of the potential therapeutic target and its validity, uncovering how to inhibit this target, while also ensuring that the solution is affordable and addressing various other considerations. Therefore, the challenges are both scientific, technical, and financial but also closely dependent on time. 

#### 2.1.1. Brief History from Ancient Time Process to Modern DD

Evidence for DD goes as far back as 2600 BC in Mesopotamia where the first drugs were based on natural products, especially plants [11]. However, much more information has been gathered today in the Ancient Egyptian area, thanks to the preservation of their writings and the Rosetta stone. The Ancient Egyptians used natural products such as calamine, ochre, and turpentine and mixed them with bread or cakes to administer the drugs [12]. In the Middle Ages, chemistry was divided into two distinct fields: alchemy and proto chemistry [13]. The first mention of alchemy dates from around 400 or 600 BC and was written in Greek; it was transcribed for the first time by the chemist Marcelin Berthelot in 1888. These writings contained alloy recipes, antique reflections towards matter and the four elements that are earth, water, air, and fire [14].

Later, one important figure of the Renaissance was Paracelsus, a Swiss physician considered as the father of “medical chemistry” [15]. He also promoted “toxicology”, and he expressed its main principles. For the first time in History, the “threshold” and “non-adverse effect” concepts were taken into account in the elaboration of a treatment [16]. 

Subsequently, DD processes have evolved alongside the evolution of chemistry. One of the key steps of this progress surely remains the extraction of pure chemical substances from plants, minerals, and animals [17]. This is the appearance of “pharmacognosy”, a science which includes analytical processes of chemistry allowing the isolation and purification of active compounds from natural sources. A historical example is the discovery of quinine. In the middle of the 17th century, Bernabé Cobo, a Spanish Jesuit’s missionary in the Andes, observed that a powder made from the bark of the Peruvian tree “*Cinchona officinalis*” was used by locals to treat malarial fevers. In the following years, this powder, called “the Jesuit’s bark”, was imported into Europe where it was progressively used by physicians. The extraction of the corresponding active substance, quinine, was performed two centuries later by the two French pharmacists Joseph Pelletier and Joseph Caventou in 1820 at the Faculty of Pharmacy of Paris. Then, the chemical structure of quinine was solved in 1854 by the German chemist Adolf Strecker, and the treatment of malaria with this drug was pursued up to the beginning of the 21st century. Among the other well-known drugs directly extracted from natural sources and discovered at the beginning of the 19th century, we must mention morphine issued from opium (published in 1815 by the German pharmacist Friedrich W. Sertürner), strychnine isolated from the “*Strychnos nux-vomica*” tree in 1818 by Pelletier and Caventou, and the discovery of papaverine by Georg Merck in 1848 [18].

Advances in organic syntheses along the 20th century allowed chemists to modify the natural products to optimise their efficiency and/or their availability. The most famous contemporary story is the pharmacological optimisation of paclitaxel (taxol), an anti-cancer agent isolated from “*Taxus brevifolia*” by Monroe Wall and Mansukh Wani in the 1970s. This molecule, which induces *in vivo* a dramatic regression of different tumours, suffers from an insoluble lack of natural availability. Paclitaxel is extracted in low yields from the bark of the tree. This process induces cutting the adult tree, which is very detrimental [19]. The French chemist Pierre Potier revolutionised the medicinal use of this family of compounds by synthesising docetaxel (patented in 1986), a molecule structurally related to paclitaxel. This new molecule was obtained starting from 10-deacetylbaccarin-III, a natural compound isolated from the needles of the “*Taxus baccata*” tree, which are regenerated yearly. In addition, biological studies revealed the high anti-cancer potential of docetaxel. Altogether, these two facts (easy renewal of the raw material and biological effects) made docetaxel one of the most popular anti-cancer agents at the end of the 20th century [20,21].

However, it is not possible to conceptualise the history of DD without mentioning serendipity. Serendipity comes from the Persian tale *The Three Princes of Serendip*, in which the Princes were always discovering things by accident [22]. An interesting fact that relied on serendipity is the discovery of the first antibiotics at the beginning of the 20th century. The discovery of penicillin by Ernest Duchesne and later by Alexander Fleming (1929) was seen as a coincidence and the factor of coincidence must be considered when dealing with the evolution of DD. On the other hand, the first sulfonamide (Prontosil) was initially discovered and used as a stain during research for antibacterial agents at Bayer in 1932. This molecule ended up protecting the mice that were infected leading German bacteriologist Gerhard Domagk to do further works on it including on his daughter. Finally, the French chemist Ernest Fourneau pursued Domagk’s work and discovered that the antibacterial agent was sulfanilamide in 1935 [23].

Another pillar of DD is the ability to create hypotheses from observation, and one of the bests examples is the progress on diabetes made by the German physician Oskar Minkowski. The link between the pathology and pancreas that led to the final discovery of insulin is said to have been discovered after the presence of flies around Minkowski’s dog, which had an ablation of pancreas. This stimulated Minkowski’s curiosity, which resulted in him tasting its dog’s urine and realising it was sweet (1889). This led Minkowski to the conclusion that the pancreas was secreting some substance that regulated the blood sugar level [24].

Modern DD started when chemistry concepts were evolved enough to be synergistically used in combination with other fields such as biology, pharmacology, physiology, medicine, and so on [17]. The combination of such sciences allowed the nuanced understanding of the disease’s process. The German scientist and 1908 medicine Nobel Prize winner Paul Ehrlich called the search for a new hit the search for a “magic bullet” [25]. His revolutionary concept may be summarised as the following: a disease results from the specific malfunction of a specific target, which may be selectively controlled by an artificial drug (the “magic bullet”). This led to our current modern therapeutic agents, able to tackle efficiently one or several human enzymes or receptors, to interfere in protein–protein interaction (PPI), to disrupt gene translation, and able to efficiently kill pathogens (bacteria, viruses, fungus…), with limited risks and side effects. 

Thus, through the ages, the DD process switched entirely. The medicine of Ancient Ages was based on the observation of the plants’ effects on the body without understanding the pathology while our modern sciences allow a precise understanding of the disease processes, leading in turn to the search of original, specific, and non-natural therapeutic agents. 

#### 2.1.2. Market: Economic Environment of DD

The DD process is embedded in a particular global economic environment. To understand the challenges of new technologies in DD, it is essential to be able to grasp the main features of the economic context in which DD evolves. First, the DD process is said to be very costly and time consuming [1,26,27,28]. A lot of studies have been conducted about the cost of the discovery and development of new medicines. In big pharmaceutical companies, the cost of DD and development are mainly held by the R&D department. If we divide the R&D cost into three parts that are the discovery, pre-clinical development, and the development process, the results show that development accounts for 50 to 58% of the total cost [28]. Depending on the study, the cost for drug R&D varies between USD 161 million and USD 4.54 billon [29]. Not only is the R&D investment required to produce a new drug huge, but it is increasing through time; the mean R&D cost has increased 43% between 2009 and 2018 [27].

However, this must be taken with caution. Indeed, there is a big impact of the Cost of Capital, which accounts for 33 to 51% of total R&D costs [28], and which will probably continue to grow as it is partially linked to inflation [30].

The 14 leading pharmaceutical companies including Pfizer, Novartis, and Roche invest a lot on R&D, and a positive correlation has been established between the number of New Molecule Entities (NMEs) and R&D expenditures: to generate 25 NMEs, an additional 16,315 employees are needed [31]. Another criterion to consider is the fact that mergers and acquisitions and in-licensing account for almost 50% of NMEs’ approvals [28]. Therefore, it is much more complicated to evaluate the real cost of DD. Moreover, a phenomenon must be considered which is called the Eroom’s law. This law states that the number of NMEs per USD billion in R&D spending is decreasing over time [32].

### 2.2. Modern DD

**Figure 1:**
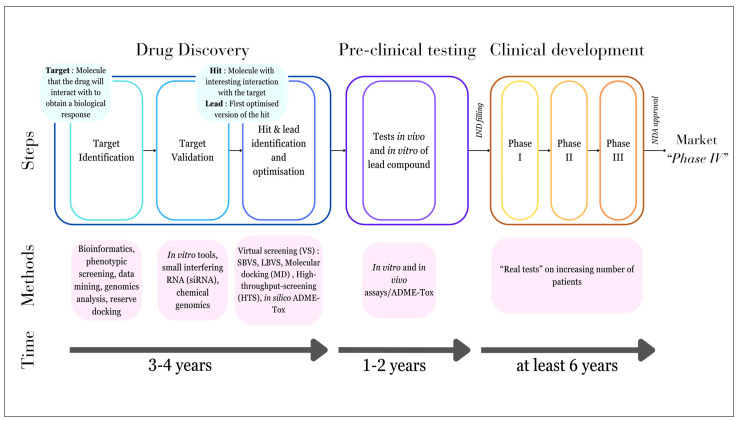
Modern drug development timeline based on the Food and Drug Administration (FDA) standards. Graphical representation of the modern drug development including the different parts from DD to the “Phase IV”. IND filing stands for Investigational New Drug Application is the request for approval from the FDA to administer an investigational drug to humans. NDA approval stands for New Drug Application’s formal proposal submitted by drug sponsors to the FDA, seeking approval for a new pharmaceutical to be marketed and sold in the U.S. Phase I: first-time drug administration to a small group of humans and assessment of safety, establishment of safe dosage range, and identification of side effects. Phase II: the drug is given to a larger group to evaluate effectiveness and verify safety. Phase III: the drug is administered to large groups of people to confirm its effectiveness, monitor side effects, compare it to standard treatments, and gather information to ensure its safe use. Phase IV: post-marketing studies are conducted after FDA approval to provide additional information about the treatment or drug, including its risks, benefits, and optimal usage [39].

DD is a costly and time-consuming process that has also been driven by luck and curiosity. With the evolution of new technologies, new processes have been created and are now used for DD. These processes involve the use of computers; this is known as computer-aided drug discovery (CADD) [33,34,35,36,37]. The goal of CADD is to allow the discovery of novel scaffolds through the use of computers and information regarding pharmacological and biological properties of potential drug targets [37]. CADD involves different methods such as *in silico* or virtual screening (VS), molecular docking (MD), Quantitative Structure-Based Relationship (QSAR), and pharmacophore modelling (PM). It also relies on the choice of approach, which could be structure-based DD (SBDD) or ligand-based DD (LBDD) [36]. Modern DD follows different steps that are presently well defined, and these methods allow for an easing of these different steps using systematic methods [38]. Here, we present a timeline of drug development that contains DD with the different modern methods involved (Figure 1). 

### 2.3. Arrival of AI and Opportunities

Even though modern DD created a more systematic way to go through all the process of DD (target identification and validation, hit and lead…) there are still some limitations. While the duration of the process has decreased, it remains long. Moreover, due to the high rate of failures during clinical phases, there is a need to find promising DCs. This can be explained by numerous reasons. In 2003, half of the drugs on the market suffered from ADME-Tox issues, and half of the DCs failed because of ADME-Tox issues [25]. In addition, a study from 2015 suggests that non-clinical toxicology is the main factor for the attrition rate in drug development from candidate to phase IV [40].

A closer look at more recent numbers puts forward the fact that 90% of DCs fail during the clinical phases and it is even more if we consider the pre-clinical phase. The main reasons are poor clinical efficacy and unmanageable toxicity [41]. Also, another known issue is the big attrition rate that happens in modern DD while using high-throughput screening that only allows researchers to keep one viable hit out of millions of candidates [42]. This screening process could be enhanced by AI [43]. 

The following section is dedicated to AI and will help the readers grasp its history, current uses in all fields, and part of its functioning. It is interesting to see how AI can help with DD and how it can provide relevant DCs. 

## 3. Artificial Intelligence

### 3.1. History of AI

The recent release of ChatGPT (2022) made AI available and tangible for the whole population, especially the generative part of AI; this has rendered AI more and more popular. According to a survey conducted in January 2023, the market size of AI is predicted to increase thirteenfold, from approximately USD 142 billion in 2022 to nearly USD 2 trillion by 2030 [44].

However, AI’s development is not so recent, and this part puts forward a brief history of AI in order to give some context of what AI is and how it has evolved.

#### 3.1.1. From 1943 to 2010

**Figure 2:**
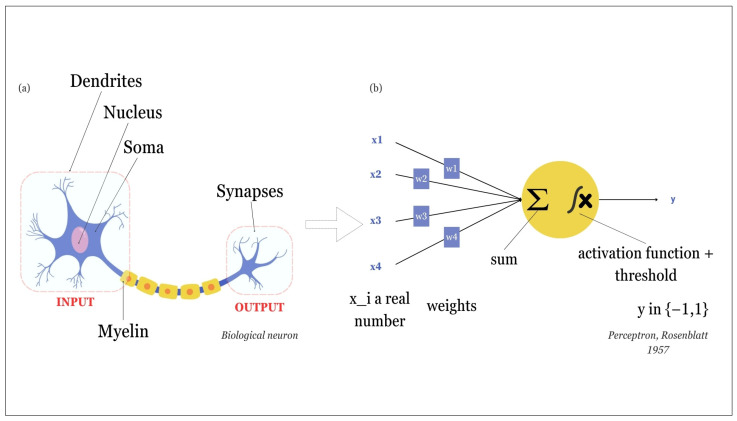
Artificial adaptation of a biological neuron. (**a**) Representation of a biological neuron as known today. (**b**) Common schematic representation of the Perceptron created by Rosenblatt in 1957.

AI as we know it today has gone through a lot of different forms. AI is based on different fields that are complementary and integrated such as mathematics, philosophy, economics, and so on. Obviously, the recollections of the rules underlying the decision process, that was first formulated by the Greek philosopher Aristotle, dated from approximately 350 BC are important. However, we chose to start our story with the birth of neuroscience and the first mathematical model of neuron which contemporary AI relies on (Figure 2).

**Figure 3:**
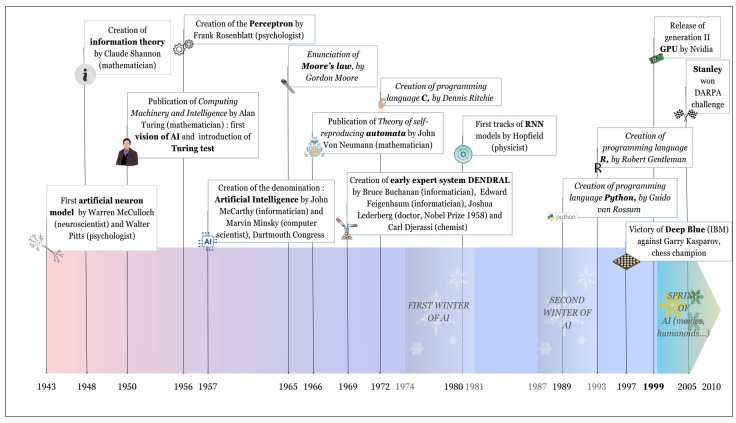
History of AI from 1943 to 2010. Chronological representation of the different events in the history of AI from 1943 to 2010.

In the 19th century, thanks to the two 1906 medicine Nobel Prize winners Camillo Golgi and Santiago Ramón y Cajal [45], the first neuronal structures became accessible (Figure 2a). Based on these works and on other theories of logic and calculus theories, Warren McCulloch and Walter Pitts [46] created the first model of an artificial neuron, that was primarily based on binary information: the neuron was either active or not (Figure 2b). This model was then modified by Frank Rosenblatt, who created “the perceptron” in 1957 (Figure 3) [47]. The first to give a full vision of AI was Alan Turing in 1950 with his confrontation work between AI and human intelligence [48]. There are numerous other famous actors in the history of AI that foresaw the AI we know today. Among them, Marvin Minsky and John McCarthy were the first to use the words “Artificial Intelligence” at a conference in Dartmouth [49] during which a lot of prominent AI actors met, including Claude Shannon, father of information theory.

The first AI that was created to simulate cognitive mechanisms for specialised tasks were known as “expert systems”. One important example is the DENDRAL project that began in 1965 and was made possible thanks to the use of a novel technique at that time: mass spectrometry. DENDRAL’s goal was to be able to analyse the chemical structure of a molecule [50].

Unfortunately, AI encountered two phases where its development slowed down: these two periods are called “the winters of AI”. The first one took place from 1974 to 1981 and the second one from 1987 to 1993 [51]. The reasons of these winters are still debated; however, it is possible to find some explanation. The AI winters came from investments cuts in research, leading to a slowdown in the development of AI technologies, exacerbated by the lack of success from expert systems [52]. Another thing said to have held back the development of AI is the lack of power of computers at the time. In 1965, Gordon E. Moore postulated that the number of transistors on a micro-processor would double every year. This became known as Moore’s law [53]. This law has been verified along time and we can put forward that in 1974, the Intel 8080 counted 6000 transistors [54], while in 2022, the Apple M1 Ultra processor counted 114 billion transistors [55]. Thus, the number of transistors on a processor or what we generally call “Central Processing Unit” (CPU) has been multiplied by around 19 million in nearly 50 years! This deeply enhanced the possibilities and the capabilities of computers leading to easier AI development.

Another important event is the release of the generation II “Graphics Processing Unit” (GPU) by Nvidia in 1999 [56]. Even though GPUs existed before, these ones were much faster. GPUs allow one to make more calculations in parallel leading to better execution and power than CPUs. This has revolutionised the field of AI allowing to test the theories that were previously created.

AI’s popularisation definitely started at the end of the 20th century. In 1997, the IBM company highlighted the potential of AI with the development of the computer called “Deep Blue”. Deep Blue was created and designed to play chess and it defeated Garry Kasparov, the famous six-time world chess champion in 1997. This first victory of a computer against a “human brain” induced a real tsunami on the publica perception of AI [57]. Between 2000 and 2010, some movies were created about AI such as *A.I.* in 2001 directed by Spielberg [58] and *I, Robot* in 2004 directed by Alex Proyas and inspired by Isaac Asimov’s *I, Robot* fiction collection [59]. It was also the advent of humanoids such as ASIMO in 2000 [60]. There was a keen interest about AI all around the world. In 2005, a team from Stanford university participated in the Defense Advanced Research Project Agency’s (DARPA) Grand Challenge, which is a challenge that asks for participants to create an autonomous vehicle able to finish a 175 miles long course in less than 10 h. The robot named “Stanley” created by the Standford’s team finished this course in less than 7 h and won the challenge [61]. Three important figures in AI who need to be cited are Yann Le Cun, Geoffrey Hinton, and Yoshua Bengio [62]. They are well known for their work on AI and Artificial Neural Networks (ANNs). Figure 3 mentions the main events of AI history with some important events to position within time such as the creation of well-known programming languages widely used today like “C” [63], “Python” [64], and “R” [65], along with some key characters’ works such as John von Neumann’s [66]. 

#### 3.1.2. From 2010 to Today

**Figure 4:**
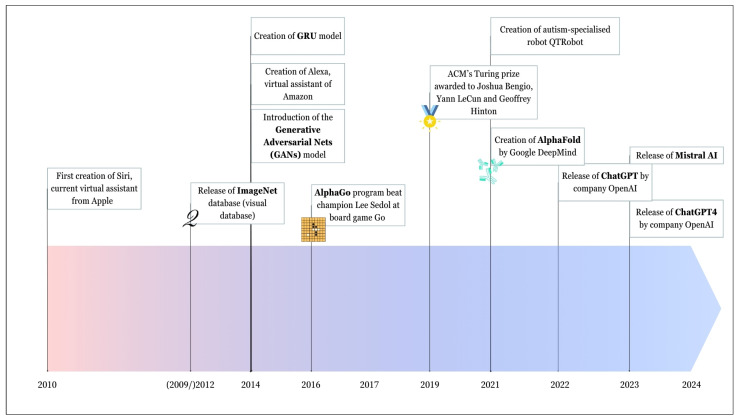
History of AI from 2010 to today. Representation of a part of AI-related events from 2010 to today.

The 21st century is witnessing a lot of the evolution of AI that has been driven by the increasing processing power of computers (Figure 4). Computers went from processing 1 to 10 million instructions per second in 1970 to processing up to 1 trillion instructions per second by 2020 [67]. This evolution enlarged the possibilities of AI. A lot of models were created to overcome the limitations of the previous ones such as transformers in 2017 in Natural Language Processing (NLP) or Generative Adversarial Nets (GANs) in computer vision. Cao and co-workers published an interesting article about the evolution of generative AI (GenAI) [68]. Transformers are based on what we call “attention”, which was introduced in the article “Attention is all you need” [69]. Attention is useful for NLP and computer vision tasks, and its principle relies on the selection of certain parts of the input data that are considered more relevant and therefore receive a higher weight compared to other parts of the input. 

Similar to Deep Blue, another event demonstrated the ability of AI to outperform humans at games by mimicking human reasoning. AlphaGo, an AI tool developed by the company DeepMind (London, UK) to play the board game Go, succeeded in beating Go champion Lee Sedol in four games, out of five matches played, in 2016. This program was based on a Monte Carlo search-tree enriched with a Deep Convolutional Neural Network (CNN) and Reinforcement Learning (RL) [70]. AlphaGo has had a winning rate of 99.8% [71]. To explain why this was an upgrade compared to chess, we must point out that a Go board game’s tree complexity, that is the search space expected in a game, is 1 × 10^237^ times bigger than that of chess [72] leading to much more complicated algorithms. This explains why AlphaGo was an indisputable upgrade compared to Deep Blue.

Through the years, a lot of new applications of AI and GenAI have arrived: First, the assistant “Siri” for Apple (Cupertino, CA, USA) in 2010 and “Alexa” for Amazon (Seattle, WA, USA) in 2014. In 2019, “Mandy.AI”, a diagnosis tool based on AI was disclosed. In 2021, “QTRobot”, a robot powered by AI for children suffering from autism, was developed [73]. 

Another important event that happened in 2021 was the release of AlphaFold 2 by DeepMind, the company that created AlphaGo. Alphafold 2 was the second version of an AI-powered system that can predict the 3D structure of a protein, based on its amino acids’ sequence [74]. This second version was accurate and created a boom in this area. Thanks to AI, DeepMind created a Protein Structure Database to make these structures available, and it counts over 200 million protein structures as of today [75]. Another important release that moved the whole world was the arrival of ChatGPT in late 2022. This really put forward GenAI and was followed by a run for companies to create generative chatbots. One of the better-known competitors of ChatGPT is the French company Mistral AI that raised EUR 385 million in 2023 [76]. 

Figure 4 mentions important events that happened between 2010 and 2024; this is not meant to be exhaustive; however, it shows the relevant events. For example, the GRU model creation, discussed in Section 3.3.2, and the well-known ImageNet dataset have been added (Figure 4).

**Figure 5:**
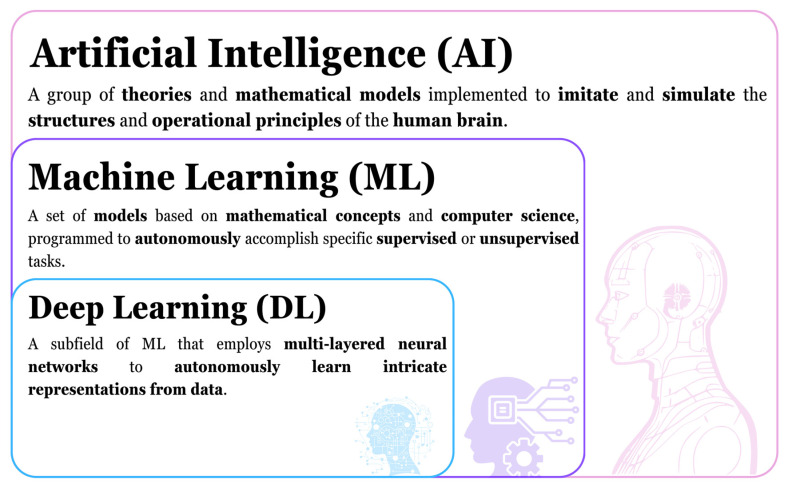
Schematic representation of AI, ML, and DL. AI is the global group that contains the two subfields that are ML and DL.

Nowadays, AI is a group of theories and mathematical models that contains the subfields of Machine Learning (ML) and Deep Learning (DL) (Figure 5). ML is a subfield of AI that relies on both computer science and mathematical concepts, it contains different models that allow for the machine to “learn”, either supervised or unsupervised. DL is a subfield of ML that mainly focuses on ANNs, which allow it to process difficult tasks. The focus here is on the use of DL; however, it is important to state that ML (without DL) techniques and DL techniques are often mixed to achieve better results and that ML (without DL) can also be used alone [77] justifying the mention of ML methods’ use throughout this review.

### 3.2. Use of AI and Emergence

“Killer app” is the name given to AI due to its ability to deeply change the way companies work [78]. Its emergence is not to be proven anymore; when ChatGPT was released, the holding society OpenAI registered over a million users in the first days of its launch [79]. AI is now used in a lot of different fields and is evolving in “Industry 4.0” [80]. AI is used mainly as a boosting tool that helps the efficiency of companies grow in accordance with the requirements of this new industry context: personalisation, flexibility, and exacerbated digitalisation. In this context, companies’ profits rely a lot on AI. For instance, companies in healthcare with a proactive AI strategy have 15 percentage points more profit than the average profit in this area [81]. Different industries are using AI such as healthcare, financial services, technology companies, or consulting companies. A publication in Bloomberg in 2022 about the rise of GenAI announced that this market could reach USD 1.3 trillion by 2032 [82].

**Figure 6:**
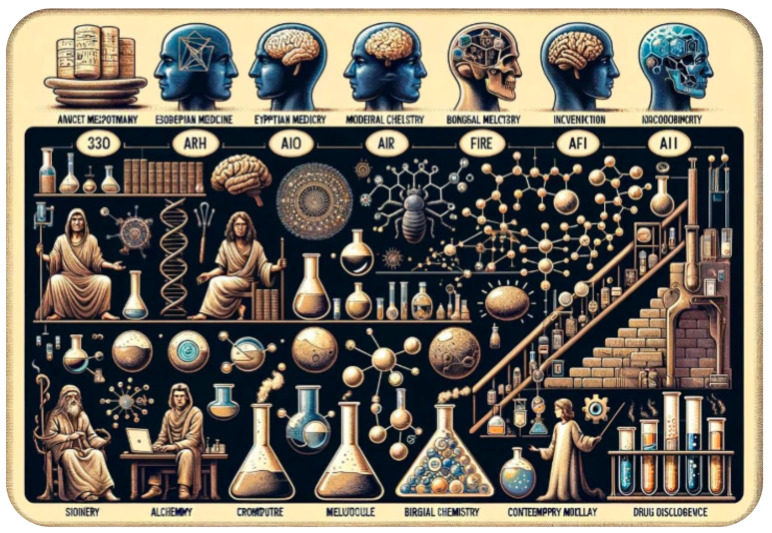
Review summary generated by AI. This image has been created using Dall-E of OpenAI based on the following prompt: “Create a graphical abstract to summarise an article on the history of drug discovery from the Mesopotamian era, through the Egyptian period, the Middle Ages with the four elements, to more modern chemistry, and onto computer-assisted discovery. Parallel this with the history of AI from biological neural networks, through the creation of microprocessors, to current models. Finally, illustrate the convergence of these two fields over the past decade and the discovery of AI-driven drugs”.

Some well-known models of AI are “Large Language Models” (LLM) such as ChatGPT used to generate text, but the company OpenAI also offers image generation with its platform Dall-E (Figure 6) [83] and realistic videos with its soon-to-be-available platform “Sora” [84]. The principle is to generate texts, images, and videos based on the user’s prompt, that is, a short or long and descriptive-enough sentence that acts as instructions for the model to generate what is asked. A new field of work has been invented, named “prompt engineering” [85,86], which is a field that encompasses the way to formulate a prompt in order to get the results that are the closest to expectations.

Some utilisations of AI in different fields can be the use of “Random Forests” (RFs), an ML model, to drive and optimise fraud detection, content editing, automation of resume selection in human resources, and so on. An IBM study has put forward that 37% of companies use AI to gain time in everyday tasks and mainly by automating content editing [87]. Regarding the expansion worldwide, an IBM’s study in 2023 reported that the countries that are deploying AI the most are, in descending order, India, UAE, Singapore, and China. The global exploration rate of AI deployment is around 40% [88].

### 3.3. AI Development and Models’ Description

#### 3.3.1. Brief Overview of an AI Development

This part briefly introduces the development of an AI pipeline to give an overview of how AI works and ease the understanding of the following parts.

The development of an AI pipeline follows different rules. From a purely practical point of view, without going into detail on the management processes of the teams involved, it is necessary to know our goal, what do we have as input, and what do we want to see as output. AI is capable of solving multiple problems, for example, regression, classification, and the generation of novel entities, among others. Once we master these sides of the issue, the next part is to know what representation of the input we will use to pursue our goal. There is already a review conducted on the different ways to represent a molecule and the different databases [9], so we do not go into detail here.

Once the input representation is chosen, the next step is to find a way to get interesting and relevant features from our input data; this can go from the number of neighbours of a node in a graph to the output of a model used as a feature extractor [10]; in the latter case, the features will be slightly less tangible. Then, the choice of the models used depends on the issue and on the input representation. For instance, if you decide to work with a graph representation of a molecule, you are more likely to use a model that can work with graphs [89,90,91,92].

The last part of your architecture is to choose how to evaluate your model. Depending on your model, you can choose among a certain number of metrics such as a ROC (“Receiver Operating Characteristic”) curve, a confusion matrix, an F1-score [93]. 

Many models have emerged from AI. One must keep in mind that there is a huge selection of models in AI going from the “simple” linear regression to autoencoders. There is a full procedure to follow when choosing an AI model for a precise issue. Something to keep in mind is that the whole architecture built when fixing a problem with AI is dependent on the skills regarding the domain. Also, ML is wider than DL, meaning that there are a lot of models that are used in ML that are not described here, such as RFs [94], “Support Vector Machines” (SVMs) [95], “Logistic Regression” [96], “Naïve Bayes” (NB) [97], and many others. The following models’ examples were chosen for the fourth and the fifth parts of this review; they present three well-known models to demonstrate the principle of an ANN. 

#### 3.3.2. “Recurrent Neural Network” (RNN)

Among all ANN models that exist, one of the most used models in DD and especially for generative DD is the RNN [98,99,100,101,102,103,104].

**Figure 7:**
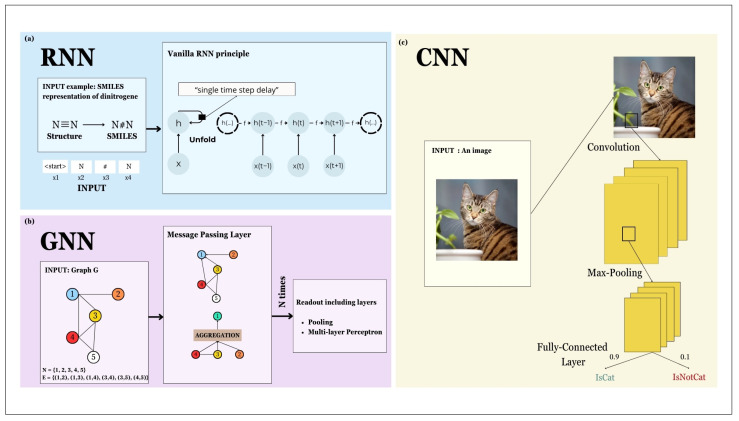
Simplified representation of well-known AI models. (**a**) Representation of the principle of the RNN model with an example of a SMILES input; the fact that the RNN is recurrent is emphasised by the unfolding of the single time step delay. (**b**) Representation of the MPNN model with a non-oriented graph input that is detailed by N and E, which are the set of nodes and the set of edges, respectively. (**c**) Representation of a classification CNN model; the cat image is filtered through a convolution layer, then undersampling is applied through a max-pooling layer, and finally, the binary classification is provided through a fully connected layer. Normally, CNN models have several convolution and max-pooling layers.

An RNN is an ANN, which means its structure is composed of different layers of artificial neurons (Figure 7a). The first traces of RNNs date from around 1982 (Figure 3) [105]. An RNN is meant to be able to manage inputs of variable lengths that can be sequences of words or time-dependent sequences like time series. The main advantage of an RNN is the fact that it considers the previous inputs through a “memory”, a so-called “state”. This state captures the past by including information of these past inputs and eventually influences the output at time t. In the basic RNN model, the so-called “Vanilla RNN”, the weight matrices that are updated during training are shared between the different inputs, the different outputs, and the different hidden states. In other words, the weight matrix for the input at time t is the same as the one for the input at time (*t −* 1). The hidden state (*h*) at time *t* can be written as follows:

(1) $$h^{<t>}=\varphi \left(W_{x}x^{<t>}+W_{h}h^{<t-1>}+b_{h}\right)$$

where $h^{<t>}$ is the hidden state at time *t*, $W_{x}$, $W_{h}$ are the weight matrices associated with input and hidden states, respectively, $x^{<t>}$ is the input at time *t*, φ is the activation function, and $b_{h}$ is the bias.

One limitation of this model is the fact that the activation function (φ) that is used most of the time is a hyperbolic tangent. Since this function is often oscillating near zero, it tends to make the gradient disappear, leading to the loss of **previous** information. To overcome this issue, two RNN variants were introduced namely “Long Short-Term Memory” (LSTM) and “Gated Recurrent Units” (GRU).

The LSTM model was proposed in 1997. LSTM can be with or without a forget gate and with or without a peephole [106]. Regardless of the variants, the goal of the LSTM model is to provide a network that is able to maintain useful past information consistently and without losing it through time. The principle of the LSTM model is to introduce a cell state inside the network. This cell state indicates to the following part if the previous inputs are relevant enough to be kept. To do so, the relevance of these inputs is summarised in the cell state. GRUs were created in 2014 [106], and they were comprehensively reviewed by Yu and co-workers in 2019 [106].

#### 3.3.3. “Graph Neural Network” (GNN)

A GNN is another type of ANN created to analyse graph inputs, and it can highlight the relationships between different nodes.

A graph is composed of nodes and edges. To give an example of inputs that can be represented as a graph, we can look at social network graphs. In these graphs, a node can be a person and an edge is created between two nodes if the two people represented by the nodes are friends. We can also have a directed graph in which an edge goes from node_1 to node_2, if node_1 follows node_2 on the social network. To give a more chemical example, we can deal with what is called “molecular graph”. This describes a molecule through the creation of a labelled graph in which a node represents an atom and an edge the bonds between two atoms.

A GNN therefore takes graphs as inputs. To base our explanations on scientific articles, we selected some articles dealing with GNNs [107,108,109]. To explain how the GNN model works, we set some notations that remain throughout this section. Let ***G*** be a graph that is defined as a pair (***N***,***E***), where ***N*** is the set of nodes and ***E*** the set of edges. The goal of a GNN is to find a function f that maps (***G***,***n***), ***G*** being the graph and ***n*** a node of the graph, into an **m**-dimensional Euclidean space [105].

There exist different types of GNNs: “graph-oriented” and “node-oriented” [107]. Most GNNs use what we call “Message Passing Neural Networks” (MPNNs). In simple words, the principle of the MPNN is to update the features of a node by aggregating the features of its neighbours [109]. If, for instance, we want to classify a protein according to its molecular graph, an MPNN can be used. It aggregates the information of the neighbours for each node, adds it as a feature to the node, and displays the result after N times (Figure 7b). This result is used to classify the graph. Some well-known examples of GNNs are “Graph Convolutional Networks” [110], “Graph Attention Networks” [111], and “GraphSage” [112].

#### 3.3.4. “Convolutional Neural Network”

A CNN is one of the best-known ANNs; this network is mainly used for image classification and object recognition.

When we deal with inputs like images, they are represented as a matrix of pixels meaning that the size is dependent on the resolution of the inputs. A CNN is fragmented into different layers in which different operations are applied. One of these operations, giving its name to the model, is the convolution. In signal processing, a convolution is an operator that is defined as follows:

(2) $$\left(f*g\right)\left(x\right)=\int_{-\infty }^{+\infty }f\left(t\right)g\left(x-t\right)dt$$

where * is the convolution operator, and *f* and *g* are two real or complex functions.

When we have a “convolution layer”, the image is submitted to a filter that transforms the initial image into a new version. There exist different types of filters, such as the “Sobel filter” that obtains the outlines of an image, or the “Gaussian filter” which is widely used to reduce the noise of an image.

A convolution layer includes different parameters: the number of parallelized convolutions, the kernel size, the padding, and the step. At the end of a convolution layer, we obtain convolution maps.

Another important layer is the “pooling layer”. The pooling layer is used to reduce the size of an image. This is mainly used between convolution layers to reduce the size of the outputs. The principle is the application of a filter throughout the image [113]. There exist different types of pooling such as “Max Pooling” which selects the maximum value above different adjacent values. Other layers are possible such as fully connected layers (Figure 7c).

There exist a lot of other models that are not detailed here to not overwhelm the article and because the best-known models are introduced here. However, alternative models are important for DD and are widely used, such as RL [114], “Variational Autoencoder” (VAE) [115], GANs [116], etc. 

## 4. AI and DD

AI, as it has been defined, is a potential asset for DD. Indeed, the use of AI could provide interesting solutions for DD from the analysis of drug–target interactions (DTIs) to optimise DCs.

### 4.1. Scientific Challenges of AI

DD is a long and costly process. The advantages of AI are numerous from cutting costs to drastically reducing research time. However, there are also challenges for AI to be consistent enough.

First, one of the main challenges in AI, especially for DD, is the availability of consistent and suitable data in huge proportion [117]. Indeed, it is essential for an AI model to be fed with enough quality data. Even though there is no real consensus to attest the quality of data, some techniques exist such as business intelligence models or statistical analyses. In health, data quality issues are social, economic, technical, and methodological [118]. In other words, there is a need for a suitable framework to manage and guide through standards health data quality. Data also need to be representative and consistent in order to provide the model with consistent explainable features. 

Another issue related to data is their availability. Indeed, even though data quality is of major importance, their quantity is also at stake when it comes to creating AI models. Nevertheless, strategies developed by researchers to palliate the lack of available data have been disclosed. For instance, for AI applied to PROTACs (Section 4.1), researchers needed to compensate for the lack of available PROTAC data. Therefore, they used quasi-PROTAC molecules, which are molecules similar to PROTACs [119].

Another promising strategy is the use of “synthetic data” (SD). SD are not based on real-world events but are artificially generated. SD can be perceived as promising because they offer to improve data quantity. It can be used for two different tasks that are data augmentation and privacy preservation [120]. SD generation allows the provision of health data in quantities that closely resemble real-world data because it is based on real-world observations. However, there is a concern regarding information leakage and outliers. Indeed, SD needs to ensure the privacy of the source data but also to be able to label outliers that are common in health data as “false data” [121]. SD provides promising results for health data with the use of models such as HealthGAN. However, this approach remains questionable since some limitations are met, not only regarding outliers but also on underrepresented classes for which SD is not capable of grasping features. It may be possible to do so by oversampling these classes [122].

Another challenge is the ability for an AI algorithm to deal with the huge chemical space of drug-like molecules. This is much more accurate when applied to a very specific case such as in *de novo* drug design [123]. Indeed, creating models that can explore the chemical space is a very challenging task. In addition, biological complexity is hard to model. Indeed, biological interactions, which are at the core of DD, are multifactorial, leading to complicated models relying on good feature selection. One of the other issues regarding biology complexity may be the lack of understanding of biological interactions, leading to the complicated orientation of the AI models. 

Another challenge is handling a large volume of data. As mentioned above, AI requires a substantial quantity of quality data to create consistent models. However, processing and analysing this vast quantity of data demands significant computational resources. Another issue regarding AI models’ performance is the hyperparameter optimisation. Hyperparameters are the parameters used to optimise the models, such as the number of layers of a CNN or its kernel size. This process can be difficult because it requires suitable resources, and it depends largely on the task [124]. 

AI models also need to be validated and interpretable. The validation of these models needs to go through experimental validation and in the context of DD, that means *in vitro* and *in vivo* validation. This can be labour-intensive, and it does slow down the advancement of AI. Another challenge is the lack of models’ interpretability that are seen as “black boxes”. This challenge is discussed in Section 4.5.

Finally, one challenge that is of major importance is the error and uncertainty management. Indeed, when dealing with AI models, there is always a risk of error and uncertainty, which needs to be tackled even more when we are dealing with health-related data. Methods have been developed to provide a framework that allows for managing and quantifying uncertainties in AI predictions. For instance, Loquercio and co-workers provided a framework that did not depend on the task, the architecture, or the learning procedures. Their framework provided an uncertainty management tool inspired by Bayesian approaches [125]. The famous paper from Abdar and co-workers provided a comprehensive review of all the methods for uncertainty quantification including Bayesian methods, Monte Carlo dropout and Laplacian approximations [126]. 

### 4.2. AI and Medicine

Even though this review is about AI and DD, it is important to keep in mind some current uses of AI in medicine. This allows us to grasp all the possibilities afforded by AI since its introduction into medicine. A wide spectrum of uses of AI in medicine have been launched, ranging from predictive analyses to voice recognition. Here, we put forward a list of uses of AI in medicine with succinct explanations and related articles. 

An important use of AI in medicine is for disease diagnosis. In oncology, CNNs can be used for colorectal cancer to analyse colonoscopy images and classify colorectal polyps. Studies on multiparameter factors can also be conducted [127] for breast cancer, using 3D CNNs to diagnose axillary lymph node metastasis, which improves the diagnosis of experts [128]. In addition, in AI-assisted ultrasound pneumothorax, regression and classification models are used to detect and classify indications, which can support physicians and help less-experienced healthcare providers. [129]. On the other hand, AI may also be used for Alzheimer’s disease diagnoses to help find new biomarkers other than τ-related and β-amyloid biomarkers. Thus, the use of different ML models (RFs, SVMs, NB) leads to improvements in the diagnosis of Alzheimer’s disease [130]. This new approach is important as the current trend is to diagnose the disease as soon as possible to use drugs such as aducanumab to destroy pathological plaques [131]. In infectiology, AI may be used to diagnose COVID-19 based on cough recordings [132]. COVID-19 disease diagnosis was also made possible by using ML on Fourier transformed infrared spectroscopy-analysed RNA extracts. This diagnosis method using dimension reduction and classification models achieved an accuracy of more than 97% [133].

Another important use of AI in medicine is personalised treatment, also called precision medicine. This is a field of medicine that is based on the past and current state of the patient, and it includes different parameters in their current treatment. Precision medicine considers genomic, clinical, and social determinants coupled with AI [134]. In cardiovascular diseases, which are interesting as they are heterogeneous and complex [135], it helps find the best treatment from the beginning of diagnosis. In oncology, it could lead to a USD 150,000 reduction in cost per year per patient [136], and it can identify phenotype imaging linked to a special mutation, which leads to improved survival rate [137]. The use of AI-powered precision medicine helps cut costs and by tailoring the treatment to patients. it leads to better results and fewer rejects. 

Moreover, an interesting progression in robots is happening. In Section 3.1.2., we mentioned QTRobot, a robot specialised in autism. In 2021, the British company Engineered Arts created an astonishing robot, “Ameca”, powered by AI and later linked to ChatGPT, that can have precise facial expressions and communicate with humans. This robot helps us understand how far robotics has progressed [138].

In medicine also, robots have progressed; for example, robots have been developed to assist surgeons in their tasks. However, current robots’ abilities are not yet sufficient to fully assist surgeons [139]. In a recent article, a team introduced a novel co-pilot robot for bronchoscopy aiming to help novice doctors to practice it as safely as possible and compensate for the lack of experts [140]. Image-guided robots have also been created to reduce intervention time and burden for patients, powered by magnetic resonance imaging and physics models [141], which will help surgeons but needs to be introduced and developed in cooperation with them [142]. 

AI has shown significant uses in medicine from approved techniques to still progressing ones. Nevertheless, AI needs data to function. 

### 4.3. Databases

The number of biological and chemical data has significantly increased in the last decades. As in other fields, a huge investment has been made to gather a lot of data, use them, discover patterns, and develop helpful models. As mentioned in Section 4.2, this would be useful not only in DD but also in emotion recognition [143], human activity recognition [144], lesion detection and classification [145], plant classification [146], biometric recognition [147], and even in dental care through caries recognition on X-ray images [148]. The available data therefore include various types of data such as images, biometrics, molecule representation, vocal recording, and so on. The first small-molecule combinatorial library dates from 1990. Moreover, since 1992, 1250 combinatorial libraries have been created [22]. As mentioned above, AlphaFold is also a source of data with its Protein Structure Database (PDB) that includes 200 million protein structures.

One issue that every industry is facing is data quality. The quality of the data is essential to create viable models. There is a real need to reduce Digital Health data quality issues [149]. Here is a non-exhaustive list of databases that are used in biology and chemistry, notably for DD: Pubchem, PharmGKB, DrugBank… Kim and co-workers presented the different databases in their survey [9]. The ethical aspect of the collection of data are discussed in Section 4.5.

### 4.4. Versatility of AI in DD 

AI is used for numerous purposes. Here, we put forward the versatility of AI in DD and the models that are used. Most of the articles published regarding DD or other uses of DL propose a mix of different models. The objective of mixing models is to overcome the flaws or limits of some models. A lot of articles focus on interactions’ predictions such as biological target identification, molecular interaction studies including DTI prediction, but also compounds’ toxicity and new organic compounds’ syntheses. To solve these challenges, researchers mainly use a mix of different models like network-based, NLP-based [150], DL-based classification through a mix of MPNNs and CNNs [151], fusion DL which aims at merging parameters of different DL models into one [152], and CNNs [88]. Something that stands out is the fact that the choice of the model in DD should not follow a “hype” but should be made based on the best-suited model for a given issue [153]. 

**Figure 8:**
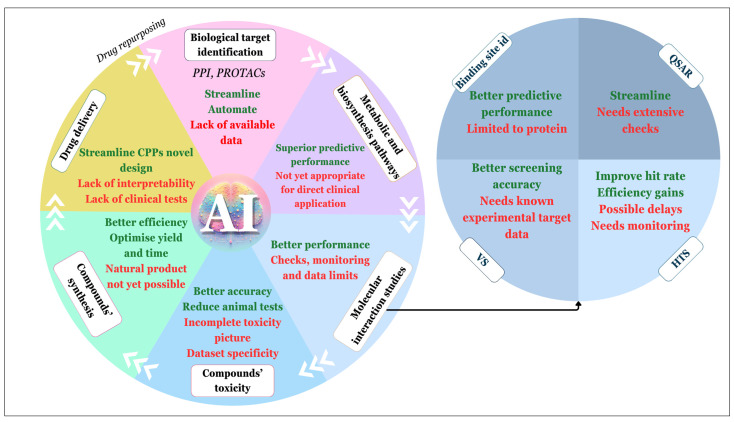
AI DD wheel. This wheel puts forward the advantages compared to conventional methods and limitations of AI in the different parts of DD. The advantages are written in green and have been gathered in the different references of Section 4.4. The limitations are written in red and put forward the current limitations of the studied models.

In order to highlight the versatility of AI in DD, this part is divided into subsections allowing us to present the contribution of AI in the different steps of DD. This part also allows us to compare conventional and AI methods and is completed by Figure 8, which exposes the advantages and limits of AI in the different fields of DD.

*Biological target identification:* Indeed, as introduced in Section 2, biological target identification is the first key step of DD. However, finding this target can be very complicated and still relies mainly on serendipity and trial-and-error processes. Target identification may result from the precise analyses and comprehension of complex interactions such as protein–protein interactions (PPIs) and/or protein–receptor interactions. Proteins have a pivotal role in biological processes, which can go from reaction catalysis to signal transduction. Signal transduction is the process that corresponds to the answer from a cell to another cell’s information, leading to a signal cascade. Therefore, the precise control of these PPIs is the cornerstone of numerous biological processes, and PPI malfunction can lead to cellular dysregulations and diseases. In turn, PPIs can sometimes represent relevant drug targets. However, PPIs are usually characterised by large hydrophobic and flat interface areas with few pockets which are not so easy to understand. The search for PPI hotspots (the amino acid residues that mainly contribute to the binding-free energy) can therefore be a really challenging task, but this remains mandatory to design small molecules (drugs) able to modify the strength of the corresponding interaction. Current conventional methods to assess this task are HTS, fragment-based DD, structure-based design, or VS [154]. Regarding the difficulties of studying PPIs, AI offers relevant methods to upgrade this task including a lot of models that are used for this task such as “Graph Convolutional Networks” (GCNs), LSTM, VAE [155], etc.

In the context of microbiology, PPIs can be seen as host–pathogen interactions. It was studied by Wang and co-workers, who disclosed a model based on a graph representation of protein structure, utilising a dual-path NN and attention mechanism. This model allowed them to predict protein–protein binding affinity and helped the analyses of host–pathogen interactions [156].

Another study that has gone through the AI-guided pipeline for PPI-based DD is the one from Trepte and co-workers. In their study, the authors identified and validated PPIs as targets through experimental and computational tools such as binary PPI assays and yeast two-hybrid screening. The use of AI allowed them to score and prioritise PPIs and to find an inhibitor of NSP10 and NSP16 interaction, which led to the reduction in SARS-CoV-2 replication [157]. 

*Metabolic and biosynthesis pathways*: When dealing with DD, the analysis and mastering of metabolic pathways such as toxic-protein degradation is of the utmost importance. PROteolysis TArgeting Chimeras (PROTACs) is one of the modern strategies used to initiate pathogenic proteins’ degradation. PROTACs are composed of a protein-of-interest (POI) ligand and an E3 ubiquitin ligase-recruiting ligand, which are called warhead and E3 ligand, respectively. These two domains are bound thanks to a chemical linker. This molecular construction triggers the POI ubiquitination by creating a bridge between the E3 ligase and the POI, leading to the degradation of the POI by the proteasome. PROTACs have major advantages from target accumulation avoiding to selectivity improvement. Unfortunately, conventional PROTACs rely on trial-and-error processes which are laborious [158]. Therefore, AI can be of huge help for this task by streamlining the process and avoiding trial-and-error processes.

Recent studies have explored the contribution of AI for PROTACs. Li and co-workers created a model that took as input an embedding of POI, E3 ligase, and PROTAC. POI, E3 ligase, warhead, and E3 ligand were represented as graphs; therefore, a GCN was used. The linker was represented using SMILES; therefore, an LSTM network was used. The concatenated inputs were then pushed forward though an MLP. This reached an accuracy of between 68 and 80% [158]. Another study from Jia and co-workers created an all-in-one platform to target protein degradation that gave better results than PROTACs, PDFBin, a nanobody-based therapeutic strategy. It could target transmembrane and solid-like disease-associated protein [159].

A promising study from Zheng and co-workers used DL and molecular simulations to generate linkers: DeLinker. They employed a strategy to palliate the lack of available data in public dataset by adding “quasi”-PROTACs molecules that were similar to PROTACs’ structure. They based their model on a sentence completion problem that is well-known in AI using a transformer, and then used RL to obtain better PK attributes. Their model had a good recovery, meaning they could reproduce well PROTACs’ chemical space, and they also experimentally validated PROTACs for cancer in 49 days [119].

Another challenging task is linked to the restructuration of biosynthetic pathways, which goes through trial-and-error processes, is resource consuming and demands time and efforts [160]. Analysing metabolism through untargeted metabolomics is also of major importance. Metabolism is a biological process that is divided into two sub-processes that are catabolism, which is the energy obtained through molecules’ breakdown, and anabolism, which lies in compound synthesis. Metabolomics is a comprehensive high-throughput approach used to study metabolism processes through the measurements of small molecules as output of metabolic pathways [161]. These endogenous metabolites (substances produced during metabolism processes) need to be captured to be analysed. AI is used in this process through untargeted metabolomics, which consists in studying metabolomics without prior biases on metabolites. AI allows one to select relevant features and identify relevant metabolites after the gathering of data through liquid or gas chromatography [161].

AI is also used to predict metabolic pathway dynamics. Conventionally, predicting pathway dynamics relies on kinetic models that needs both in-depth expertise in the form of detailed system-specific information and time. ML can be used to predict this pathway dynamic through the use of a large quantity of now-available multiomics data [162].

Jang and co-workers also explored the use of AI to construct novel biosynthetic pathways through an enzyme design approach. They created a model called RetroPath RL and a SMILES metabolite representation that could predict biosynthetic pathways from L-glutamate to mesaconic acid [163]. Another study based on the very lethal gastric cancer from Chen and co-workers explored the use of ML predictors for diagnosis and prognosis of gastric cancer. There is a need to find non-invasive diagnoses and the limits of basic metabolomics studies for gastric cancer plus the need to develop and refine metabolomic biomarkers to pave the way for the use of AI in this field. They created a model based on Lasso regression and an RF to find 10 metabolites allowing them to discriminate gastric cancer-suffering patients and disease-free patients. The model obtained satisfying results and promised to cut costs even though there is no direct application yet [164]. 

*Molecular interaction studies*: An important use of AI is protein binding site identification. Indeed, DL has been used to predict protein binding sites through models such as graph transformer-based DeepProSite, using as input a sequence representation of the protein and the protein’s structure through ESMFold, which is a pretrained protein language model that allows one to predict proteins’ structures from single sequences [165]. 

To go further in the use of AI in DD, one can mention what is called “deep docking”. Deep docking is based on a deep NN and inference. Only a fraction of the molecules is docked, and the input of the NN is composed of this molecular docking and the SMILES representation of the interacting molecule. Once it has been completed, the docking of the other molecules is found by inference [166].

Another use of AI in QSAR is the so-called “deep QSAR”. Conventional QSAR is well suited for AI as it is based on a molecule representation such as SMILES or a molecular graph. After collecting the data, conventional QSAR calculates explicit chemical descriptors. Deep QSAR is different because it creates a full embedding of the molecules, which is incorporated in the optimisation of the model, meaning that the chemical space is a part of the model optimisation [167]. Deep QSAR is useful as it can incorporate multi-objective tasks.

Something to keep in mind is that conventional computational models and DL models are used together to maximise the potential of each [168]. VS is also impacted by DL. There are different types of VS such as “Structure-Based Virtual Screening” (SBVS). SBVS is based on the optimised target’s 3D structure (identification of active sites, suppression of unnecessary water molecules…). During SBVS, the target is docked to a huge number of molecules and outputs an affinity score for each of them. AI is used for SBVS through the creation of models such as RFs or CNNs leading to the creation of ML scoring functions (SFs). This ML SFs’ training is based on specific features leading to an enhanced discriminative power compared to classical SFs [169].

Additionally, AI can also be used in already existing computational methods in order to enhance the, such as high-throughput screening (HTS). HTS is a computational, automated, and cost-effective tool that allows one to screen a huge number of molecules. Its goal is to identify promising DCs. HTS works by applying alternative screens or filters to a database of molecules. AI can be used here to improve HTS by integrating neglected aspects of the process. Indeed, HTS’s filters are of varying size and fidelity, but the information contained in both filters are not considered, such as the magnitude order between primary and confirmation screens. Therefore, ML is used to integrate into the molecule representation low- and high-fidelity measurements [170]. DL can also be used to reduce the attrition rate of HTS’s screening [171]. 

*Compound toxicity*: Compound toxicity is a major driver of DCs’ failures in DD, the main reason in 30% of cases. Therefore, there is a real need to manage toxicity and improve toxicity prediction early in the DD. Sharma and co-workers created a DL framework to improve the accuracy and interpretability of clinical toxicity predictions through a deep multi-task model. They opted for an explainable model called “contrastive explanation method”. They used two types of input, SMILES and Morgan fingerprint, and their model was based on deep NN. They obtained promising results, but they realised that *in vivo* data did not help enhance the prediction of toxicity [172].

Still regarding toxicity and compound response to toxicity prediction, Hao and co-workers created a DL model called Dtox, which aimed at deciphering cellular mechanisms of toxicity *in silico*. Their predictive model was based on a visible neural network which was based on knowledge from biological pathways. Their results were as good as those reported using models based on MLPs or RFs [173].

One important toxicity when developing a drug is the human-ether-a-go-go-gene (hERG), that is its involuntary inhibition. hERG is essential for the heart as it enables the heartbeat coordination; therefore, watching its toxicity is fundamental to reduce cardiac side effects. Therefore, a lot of models have been developed to predict toxicity, including SVMs, Deep NN, RFs, NB, ANNs, or Boltzmann machine-based models. Similar studies have been made for lethal dose, 50% and drug-induced liver injury. The results show that AI increases speed, decreases costs, and leads to better success rate [174].

*Compounds’ synthesis*: One of the most important steps after identifying a potential lead in DD is the compound’s synthesis. Organic syntheses are today limited because of the tedious reaction optimisation for single steps and synthetic route adaptation. The next step is the full automation of multistep synthesis through the creation of an AI-guided synthesis planning [175].

Organic syntheses result from retrosynthetic analyses. Retrosynthesis is the search for the most suitable synthetic pathway, using easily available and costless raw material, and limiting the synthetic steps. In addition, current retrosynthetic analyses take into account as often as possible the “12 principles of green chemistry”, which involve, for example, the use of eco-sustainable processes, the use of eco-friendly and non-toxic solvents, the use of low-energy reaction conditions such as microwave and ultrasound… Also, retrosynthesis is labour-intensive, a knowledge-driven task, and it requires a high level of experience and knowledge of the dedicated literature. To save time and energy, there is a clear need to streamline and automate retrosynthesis. AI can therefore play an important part in this need through optimising and maximising yield and selectivity, leading to better resource and waste management. The uses of AI in this field are classification models for ligand reactivity, regression models for ligand parameter correlation, MLPs for regioselectivity, RFs for enantioselectivity, and transformers for retrosynthetic pathway prediction [176].

Segler and co-workers also proposed a model based on Monte Carlo Tree Search to improve the transformations’ extraction process, which was then mainly based on algorithms that led to high noise and a lack of chemical intelligence. The challenge there was to develop a model that differed from conventional search problems, such as those used in chess. Monte Carlo Tree Search was good for large branching with few heuristics, and the authors also added three more NNs. The model showed good results and reasonable routes [177].

*Drug delivery*: DL is also of utmost interest to set up efficient strategies in drug delivery processes. For example, there is a need to create effective cell-penetrating peptides (CPPs). CPPs are peptides with the unique property of being able to penetrate cell membrane and therefore allowing the delivery inside the cell of molecular cargoes (drugs, dyes, etc.). CPPs’ creation is a laborious and time-consuming process not always leading to optimised ones. A CPP model based on DL was created to ease their design and to optimise their structure [178].

Hassanzadeh and co-workers put forward the combination of nanotechnology and AI for applications in neuroscience and cognitive biology. They proposed AI-guided microspheres that delivered the drug to the right target by analysing the activity of pathogen agents and detecting this activity through electromagnetic fields. [179]. He and co-workers used AI, more precisely a Decision Tree, an SVM, and an NN, to create specialised antibiotics that would not be general antibiotics but would specify themselves with respect to the selected target [180].

*Drug repurposing*: Finally, an important aspect of DD is the use of AI for drug repurposing or repositioning. For this kind of issue, and to find different promising drug candidates, VAE is used [181]. Autoencoder techniques are widely used in DD. It is also popular to use trained models to screen the FDA-approved drugs’ library. A good example of it is the use of graph convolutional networks to find potential inhibitors of a target among all FDA-approved drugs [182]. 

### 4.5. Ethical Limitations and Transparency

Ethical issues are another big challenge for the use of AI in DD. Indeed, the data need to be anonymized and special laws must be applied to respect privacy and human rights. Therefore, as there exist commissions and agreements to guarantee ethical practices in biology, such as the “Déclaration d’Helsinki”, the submission to ethical committee, or the written approval of patients, there exist rules and laws concerning AI, notably, regarding the gathering of data and their use.

A lot of scientists consider the dangers that could represent AI. For instance, in an interview with Toronto Life in 2023, Geoffrey Hinton expressed his worries saying, “I don’t think there’s any chance of us maintaining control if [the machines] want control.” [183]. Another well-known scientist, Yoshua Bengio, contributed to the creation of the “Déclaration de Montréal pour une IA responsable” that aims at insuring that AI is used in a proper manner and for the well-being of the population [184]. 

On a more governmental scale, the European Union decided to create the European Union’s AI Act in 2021 to regulate and create a legal framework for a suitable, responsible, and viable development of AI [185]. Among all the proposed rules, there are the prohibition of AI practices that contain unacceptable risks and the obligations to respect when deploying or providing high-risk AI applications and list these high-risk applications. If we cross the Atlantic Ocean, other tactics have been applied. The USA created the AI Safety Institute Consortium, AISIC, a consortium aiming at creating guardrails for the use and development of AI [186]. These guidelines are numerous; some of them deal with the development of tools and methods to facilitate the industry standards to develop AI safely and securely, with the creation of secure-development practices for generative AI, the implementation of guidelines for successful red-teaming and ensure privacy… An interesting article about AI’s governance is the one from Corinne Cath [187]. However, even though there is a concern about an ethical use of AI, AI can be relevant to enhance the current ethical issues that are encountered. Indeed, the use of AI applied to organ-on-chip could prevent the use of real animals. Also, AI shows promising results in reducing the use of *in vivo* experiments. Indeed, Sharma and co-workers put forward the fact that AI model results were not enhanced by *in vivo* results when using SMILES input, and that *in vitro* experiments were sufficient to obtain a sufficient area under the curve [172].

Another important issue with AI is the transparency of the algorithmics that are used. Indeed, these algorithms are often seen as “black boxes” meaning that what happens inside the NNs is not understandable and therefore may not be trustable and subject to uncontrollable biases. However, significant efforts are being made to use explainable models, such as the explainable DL model that are presented in Section 5.3. Researchers are trying to introduce this notion of explainable models as much as they can to anticipate the future management of these established models. Indeed, Sharma and co-workers used a “Contrastive Explanations Method” to explain black boxes through pertinent positive and pertinent negative features that allow them to grasp features’ properties [172].

## 5. Case Studies (Wet-Lab Validation and from Drug Candidates to Clinical Phase)

In this part, we put forward three recent case studies that highlight the use of DL techniques since 2022. This shows what is currently used in the field of AI applied to DD. Here, we present three case studies about the process of finding inhibitors using AI and one about the identification of a new structure of antibiotics. We tried to put forward case studies that incorporated results from *in silico* DD to wet-lab experiments. Thanks to that, we are able to grasp the efficacity of AI.

### 5.1. Generative DL: Receptor-Interacting Kinase 1 (RIPK1) Inhibitor 

Y. Li and co-workers [10] introduced their work on a potential RIPK1 inhibitor accompanied by AI. In that article, the authors justified their choice for the model, and they went from the AI-powered *in silico* inhibitor discovery to the *in vivo* part.

RIPK1 is a serine/threonine protein kinase that has an impact on cell necrosis. As a key regulator of programmed cell necrosis, RIPK1 takes part in the occurrence and development of inflammatory and immune diseases. RIPK1 has a central role in cell necrosis and is therefore considered as a promising target to cure cell necrosis-associated diseases. In their article, the authors investigated the discovery of an active compound with a new scaffold and proposed a validation wet-lab experiment.

They generated their own potential inhibitors’ library for the selected target using a distribution-learning conditional RNN, which is an RNN that includes initial hidden states based on prepared values, such as fingerprints or descriptors; the chosen data representation for input was SMILES (Table 1). 

The authors decided to create a model based on a conditional RNN (cRNN) with a LSTM layer. A cRNN differs from an RNN because it provides the model with an explicit initial state vector that allows the model to generate molecules into a specific domain. The model followed these steps: molecules were transformed into SMILES, then a feature extractor was applied, and a one-layer bidirectional LSTM was used to obtain the initial state vector. Next, transfer learning and regularisation enhancement were applied for the training part, and sampling enhancement for the generation part. 

Transfer learning was used in order to palliate the lack of known RIPK1 inhibitors. The goal of the training part was to train the model to reconstruct the initial molecules. First, the model was trained on a large dataset containing more than 16 million molecules, that is, the ZINC12 [188,189,190] database, and was then fine-tuned using 1030 known RIPK1 inhibitors. Transfer learning allowed them to enhance time and performance. Regularisation enhancement, that is, randomly adding Gaussian noise to the state vector, was used to enhance the generation method. Sampling enhancement, more precisely linear-interpolation sampling, was used to enhance the generation of new molecules through better random state vector sampling in the learned latent space. The built model was then used to generate a library for RIPK1 inhibitors. Then, this library went through VS to obtain drug-like hit compounds targeting RIPK1 and to generate a pharmacophore map of RIPK1 inhibitors. After some tests, eight compounds with relatively easy synthetic accessibility were chosen for synthesis and bioactivity evaluation. 

Out of the eight synthesised molecules, the authors focused on RI-962. After the determination of the co-crystal structure of the RIPK1 kinase domain in complex with RI-962, the authors proved that RI-962 occupied both the ATP-binding pocket and the allosteric site, simultaneously meaning that RI-962 was a type II kinase inhibitor. Compared to other type II RIPK1 inhibitors, due to its rotation properties, RI-962 induced a conformational change around the allosteric site. This result suggested that there was a suitable space in the allosteric site as well as enhanced interactions between RI-962 and residues in this pocket, highlighting the potential high selectivity of RI-962 for RIPK1.

Then, pharmacokinetic and safety evaluations in Sprague Dawley rats were conducted leading to a proper drug exposure, good metabolic stability, and a good tolerance for mice without side effects. Eventually, *in vivo* tests were conducted on a TNFα-induced SIRS mouse model. Without treatment, the survival rate was 10% (in 24 h) while RI-962-treated mice had a survival rate increase of 90%. Other effects were shown, such as a temperature loss, decreased concentrations of proinflammatory cytokines, and liver and kidney damage attenuation. RI-962 was also tested on DFF-induced IBD model. In both TNFα-induced SIRS and DFF-induced IBD models, Western blot assays demonstrated that RI-962 had no impact on the RIPK1 protein but substantially reduced the level of phosphorylated RIPK1 leading to the non-activation of the underlying downstream proteins.

### 5.2. Small-Molecule TRAF2- and NCK-Interacting Kinase (TNIK) Inhibitor

Another very interesting article published in 2024 [191] by F. Ren and co-workers describes the findings of a target and its inhibitor to fight against idiopathic pulmonary fibrosis (IPF). The search for such drug has become of utmost importance, since pulmonary fibrosis prevalence has increased dramatically in the context of the post COVID-19 pandemic.

The authors wanted to discover a target of interest and a small-molecule inhibitor for IPF by using predictive AI. This article is interesting because it puts forward the use of commercially available AI-driven platforms that allow for a greater ease of finding of target.

To find their target, the authors used the platform “PandaOmics” [192,193], which is partially based on pretrained transformers, and they completed the search with a method that trained the model to predict from data stopped at a precise time the targets of interest. The authors trained the AI models using different types of datasets, such as lung fibrosis data from patients (including genomics and metabolomics data), as well as text-based data (including scientific reports, clinical trials results…). They also used several filters including a “protein and receptor kinase” scenario and another to select biological targets allowing the probable selection of novel (patentable) and druggable small-sized inhibitors. This strategy allowed them to identify a short list of relevant biological targets, ranked by order of interest. Thus, AI identified the serine threonine kinase TNIK (TRAF2- and NIK- interacting kinase) as the top target. Even if TNIK has been described in the cellular signalling pathways implicated in this fibrosis, it has never been targeted itself by researchers to disrupt the pathological process of this disease.

In the second step, the authors used another commercially available platform, “Chemistry42” [194,195], to select a promising TNIK inhibitor. Taking advantage of the already available structure of TNIK, the authors decided to design an ATP-binding pocket inhibitor, which is a conventional approach to inhibit kinases. Therefore, the AI platform was conditioned to explore molecules with the capacity to form a network of hydrogen bonds with selected amino acid residues of the TNIK ATP pocket. To ensure the selectivity of the inhibitors, the AI model was instructed to select compounds with an additional hydrophobic pharmacophore able to interact with allosteric pockets, more specifically, TNIK. Finally, filters were applied to select patentable and synthetically accessible small molecules with an optimized pharmacological profile. The lead molecule, called INS018_055, exhibited very promising *in vitro* and *in vivo* results, with a marked anti-fibrosis effect in different tissues (mainly lung and kidney). Its use was validated, and the molecule entered in clinical trials phase 0 (in Australia) and phase I (two trials in China and New Zealand were reported). These trials demonstrated the safety and tolerability of the molecules and provided information concerning its bioavailability and its pharmacological profile in healthy volunteer patients. 

It must be underlined that from the beginning of the project to the preclinical phase, this process only took 18 months which is from 2.7- to 4-times quicker than traditional methods. This demonstrates unambiguously the unique potential of AI for speeding up the DD process. Nonetheless, in this case, the enthusiasm may be balanced since this case study is very favourable. The selected biological target is a kinase; hundreds of kinase inhibitors have been designed in the last two decades, and approximately 10 new kinase inhibitors have been approved yearly since 2020. This facilitates the AI learning and analyse processes (Table 1). 

### 5.3. Explainable DL: New Structure Class of Antibiotics 

After the finding of the first antibiotics by serendipity as explained in Section 2.1.1., researchers have been developing novel strategies to create a more systematic way to find antibiotics. This new way to find antibiotics is urgent regarding antimicrobial resistance (AMR), and over the past 40 years only a small number of new antibiotics were issued from a novel class [196]. F. Wong and co-workers [197] put forward the use of what we call explainable DL, that is, the use of methods that allow for the understanding of the underlying processes of a DL algorithm to discover a new structure of antibiotics. In this example, they used GNNs and decided to work with explainable AI by explaining the GNN model.

**Table 1:** Case studies table displaying the different parts of the AI-based methodology development of different articles.

Article	5.1: Li, Y. et al., 2022 [10]	5.2: Ren F. et al., 2024 [191]	5.3: Wong F. et al., 2024 [197]
**Context**	Receptor- interacting protein kinase 1 (RIPK1) a serine/threonine-protein kinase that plays a part in cell survival especially in apoptosis and necroptosis. RIPK1 was selected as a promising target for necroptosis-related disease.	Idiopathic pulmonary fibrosis (IPF): disease that leads to a high mortality rate with limited FDA-approved treatments.	In the context of the antibiotic resistance crisis, we need to discover novel structures classes of antibiotic against *S. aureus*, a Gram-positive pathogen, for the difficult treatment of nosocomial and bloodstream infections.
**Goal(s)**	Generate a small-molecule **inhibitor** against RIPK1 with novel scaffold thanks to AI.	⇾ Identify an anti-fibrotic **target**. (1) ⇾ Generate an **inhibitor** of this target. (2)	⇾ Identify novel structure classes of **antibiotics**.
**Data representation**	SMILES	Multiomics data containing different types (survival, age…)	Morgan fingerprints
**Database(s)**	⇾ ZINC12 database (ca. 16 million molecules) **SOURCE** DATA. ⇾ RIPK1 known inhibitors library (1030 molecules) **TARGET** DATA, transfer learning.	⇾ 15 IPF multiomics datasets from GEO database. (1) ⇾ Hierarchical Active Molecule (HAM) included in the structure-based platform.	⇾ Set of known antibiotics, natural products, and other molecules. ⇾ Mcule purchasable database. (a) ⇾ Broad institute database. (b)
**Model(s)**	Creation of a generative DL model based on: ⇾ Conditional RNN. ⇾ Transfer learning. ⇾ Regularization enhancement.	⇾ Predictive approach: a commercially available AI-based platform based on pretrained transformers. (1) ⇾ Generative approach: a commercially available structure-based drug-design AI platform + available crystal structures of the target kinase domain. (2)	⇾ Explainable DL using Chemprop, GNN platform + graph-based search algorithm for explainability. ⇾ Orthogonal model to predict cytotoxicity.
**Selection methods**	Once the inhibitors library had been created (ca. 79,000 molecules) it was subjected to different selection methods: ⇾ Delete same Murcko scaffolds + virtual screening: drug-like screening using pharmacophore map (ca. 23,000 molecules). ⇾ Docking + top-rank selection + interesting features selection (8 remaining molecules).	⇾ Selection of TRAF2- and NCK-interacting kinase (TNIK) through “protein and receptor kinase” approach + confirmation of TNIK potential. (1) ⇾ Selections including pharmacophore points + *in vitro* + lead optimization. (2)	Creation of four ensembles predicting antibiotic activity after preliminary training and applied to (a) + (b) (ca. 12 million molecules). ⇾ Apply ensembles (ca. 10,000 molecules). ⇾ Cytotoxicity score < 0.2 (ca. 3000 molecules). ⇾ Rationale analysis (380 molecules). ⇾ Exhibition activity against target + other inhibitory experiments (2 hits).
**Results**	Selection of final promising inhibitor: RI-962 ⇾ *in vitro*: inhibition of RIPK1 kinase activity (inhibition of RIPK1 phosporylation and dowstream signal) ⇾ *in vivo*: survival rate increased by 90% in RI-962-treated mice, RIPK1 phosphorylation rate decreased.	After selection of final inhibitor: INS018_055: ⇾ *in vitro*: selecting at inhibiting TNIK. ⇾ *in vivo*: strong anti-fibrotic phenotype for mice treated with mix therapy including INS018_055. ⇾ preclinical: dose of 100 μg well tolerated by humans. ⇾ phase I: good toleration of oral bioavailability in healthy volunteers.	⇾ *in vitro*: favourable selectivity of both compounds, not genotoxic. ⇾ *ex vivo*: non-toxic when applied topically; decrease of mean bacterial load.
**Time needed**	Unknown	18 months (from discovery to preclinical phase included)	Unknown
**Article**			

They found a class of antibiotics that worked against *Staphylococcus aureus (S. aureus)*, a Gram-positive pathogen. Each model was supplied a list of RD-Kit-computed molecular features that were resistant to many first-line antibiotics. They used “Chemprop” [198], an MPNN, to predict the probability of a molecule to be active against bacterial growth depending on its structure. This approach of a DL-guided process used the antibiotic potential and the cytotoxic profiles (experimentally obtained) of 39,312 molecules to “train” the AI model. Then, the resulting GNN was applied to a library of 12,076,365 commercially available molecules. This allowed the researchers to classify these molecules thanks to a rational analysis of “predicted” structure–activity relationships; finally, a chemical class of molecules combining both an antibiotic activity and a low cytotoxicity emerged, and two hits were identified (Table 1).

This method also differed from traditional methods because their work was based on the explainable aspect of their model. Indeed, they made the bet that they could find a new structural class by exploiting the substructures on a compound hit and chemical substructures. This study shed light on the capabilities of AI to change antibiotic research methods but also on two advantages of explainable DL, a better understanding of the model and more transparency. Also, this model surpassed conventional methods such as QSAR as it did not require any prior knowledge of active scaffolds. 

This new antibiotic harboured an unrelated, yet effective, chemical structure. Thus, it provided to this antibiotic significant Gram+ specificity and strong efficiency against antibiotic sensitive *S. aureus* and resistant mutant *S. aureus*. In particular, this new compound family was able to act on methicillin-resistant *Staphylococcus aureus* (MRSA). Currently, MRSA may be treated by vancomycin from the glycopeptide class, daptomycin (Cubicin) from the lipopeptide class, linezolid from the oxazolidinones class, ceftaroline (Zinforo) from the cephalosporin family, dalbavancin (XYDALBA) from the glycopeptide class and lysocin E [199].

However, some resistance appeared for vancomycin [200], ceftaroline (reduced susceptibility) [201], linezolid (0.1% resistance at this time) [202], or some inefficiency events such as with daptomycin (inactivation by surfactant-22) [203]. Due to the adaptability of bacteria, fighting infection constantly required new classes and modes of action.

The most conventional basic mechanisms of antibiotic action against bacteria consist in the inhibition of cell wall synthesis (most common mechanism), inhibition of protein synthesis (translation) (second largest class), alteration of cell membranes, inhibition of nucleic acid synthesis, and antimetabolite activity. However, MRSA strains are tolerant of conventional antibiotics but sensitive to the new major antibacterial key, the proton motive force (PMF), conferring it the status of new treatment strategy to eliminate antibiotic-tolerant cells [204]. Here, this newly AI-generated antibiotic targeted this specific way thus providing its bactericidal potency via PMF inhibition by disrupting the proton gradient (ΔpH). Nevertheless, other chemical antibiotic agents including nordihydroguaiaretic acid, gossypol, trifluoperazine, and amitriptyline which strongly disrupt the PMF in MRSA cells by dissipating either the transmembrane electric potential (ΔΨ) or the ΔpH are also under evaluation [204].

Previous studies have indicated that the uptake of tetracyclines by bacteria depends on ΔpH, whereas aminoglycosides use the ΔΨ.

Taken together, the AI development of antibiotics brought a new unrelated antibiotic family for MRSA treatment through ΔpH. 

## 6. Discussions

**Figure 9:**
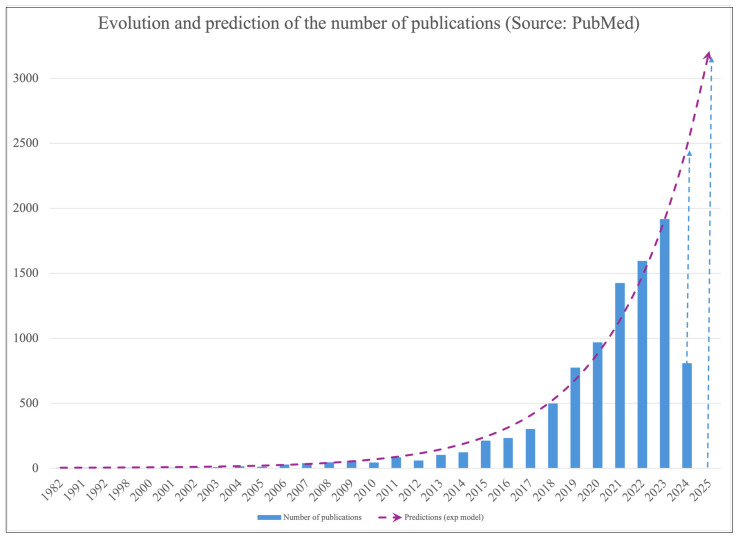
Evolution of the number of publications for the keywords [(machine learning) AND (drug) AND (discovery OR development)] in PubMed and exponential model prediction for the upcoming years.

DD has a development story stretching back millennia, from the Mesopotamian era based on natural products to modern practices including the use of AI. This rich story sets the foundations of DD from observation and serendipity to a more systematic analytical and scientific way to discover drugs. AI’s history began around 80 years ago with the arrival of the first artificial neuron model mimicking the structure of the biological neuron. AI’s involvement in DD processes started at the beginning of the 21st century; its craze in DD can be easily demonstrated through the evolution of the number of publications regarding ML and DD in PubMed, highlighting that we are at the beginning of the exponential evolution, a real “Big Bang” (Figure 9).

Nowadays, AI models are far more advanced and allow numerous operations from classification to generation, with the well-known LLM ChatGPT that created a real awareness among people as to what AI can now do, which is far from the first deterministic chatbots. Along the beginning of the 21st century, a lot of advanced models emerged from GRUs to the different versions of GPT. A craze emanated from ChatGPT, and companies have started to invest a lot in AI to automate time-consuming tasks. There is a particular focus on generative AI, and these models are now used and applied to DD. RNNs, CNNs, GNNs, VAEs, GANs are now used to generate novel scaffolds for inhibitors, drug-target prediction, drug repositioning, and so on. The time-consuming and costly process that is DD leads to a promising time ahead for AI on which a lot of hopes rely. To help scientists in the search for new biological targets and their specific modulators, there exist a lot of commercially available platforms that allow them to both discover a target towards a special disease and generate interesting hits. One of the most relevant tasks in the DD process in the future will surely be to properly select the right AI platform to design a molecular lead, depending on the pathology and/or target studied. 

Thanks to these advances, we reported in this review the discovery process of a molecule selected to cure idiopathic pulmonary fibrosis. In this study, AI platforms were everywhere, since they helped with the identification of the biological target (TNIK, a serine-threonine kinase), the molecular hit selection, and the subsequent “hit-to-lead” optimisation processes. Indeed, two AI platforms were sequentially used (PandaOmics for the target identification, then Chemistry42 for the chemical lead identification), and the selected molecule successfully passed *in vitro* assays, *in vivo* assays, pre-clinical, and clinical trial phase I, in only 18 months, and have reached now the phase II clinical trials. This is a spectacular success story, which undoubtedly highlights AI’s efficiency in speeding up DD from the target selection to the design of non-toxic and efficient molecular lead. Of note, this performance may be balanced by the fact that since the commercial release of imatinib, the first kinase inhibitor used clinically from 2001 to treat leukaemia, kinases have been subjected to numerous studies of medicinal chemistry. In 2020 alone, 10 kinase inhibitors were clinically approved! Thus, in this case, AI learning is simplified, thanks to a large set of available experimental data. Learning will be much more challenging and time consuming in more complex, less-explored cases, such as the modulation of PPIs by small-sized organic molecules. 

Another relevant result highlighted in this review is the AI-mediated discovery of a novel class of antibiotics. That study may be considered as a deep learning-guided approach, since it used 39,312 molecules, experimentally studied for their cytotoxicity and antibiotic profile, to train the AI and generate the graph neuronal network. Applied to a library of 12,076,365 molecules, the AI platform led to molecules able to deregulate the ΔpH and specifically active against the MRSA stain. This is definitively a breakthrough for one of the most important scientific frontiers in medicine, since no new classes of antibiotics had been reported in the last 40 years! This situated itself in an antimicrobial resistance context which brought this discovery to the public. The selected hit already proved its efficiency *in vitro*, and its non-toxicity. However, this story is less advanced than the previous one, and the next step is to determine if this promising molecule will reach the clinical trial stages or not. This promising study on antibiotics’ structural class research allows for the completion of the previous seminal paper that set the foundations for the use of DL in antibiotics discovery [205]. In this paper, the authors used a DL-based model composed of an MPNN (Chemprop) and the “RDKit” [206] and discovered a molecule, halicin, which was structurally divergent from conventional antibiotics, had bactericidal activity against a wide range of pathogens, along with eight additional antibacterial compounds with structures distant from existing ones.

Fortunately, AI will make even more progress leading to a better understanding of DD mechanisms. However, as with the issue of HTS attrition, there is an issue with generated molecules. Indeed, AI can generate a lot of molecules, but only a few of them are considered as promising candidates. Therefore, we could ask why AI is not yet able to generate dozens of promising molecules when it comes to generating inhibitors against a target, all the more as when compared with the remarkably high AlphaFold accuracy. We can suggest two reasons for this. The first one could be that AlphaFold generates 3D-structures based on amino acid sequences and was trained on a lot of available labelled data. The chemical rules responsible for the secondary, tertiary, and quaternary structures of a protein have been well known for decades and perfectly elucidated and/or generated from the amino acid sequences. They are based on electrostatic interactions and hydrophobic effects which can be learned by AI. Therefore, a 3D model of the protein created by AI has to understand the folding of proteins but takes advantage of a supervised and well-documented manner to do it. As a marked contrast, the identification of a peptide’s inhibitor or of a PPI’s modulator is much more challenging for AI. Most of the time, the models to learn are based on the auto-encoding of molecules and then a fine-tuning on known inhibitors that can be scarce. This may be linked to the history of DD, which is a mix of different fields and includes a huge part of exploration. Moreover, the structures of the targets differ strongly and the number of chemical spaces around the potential small-sized molecular inhibitors is infinite. Even if several interactions or active-site structures are well documented (e.g., kinases), the level of complexity and freedom is much more important than those encountered for the elaboration of a 3D structure of a protein by AlphaFold. Nevertheless, the race for computer power in the era of processing power-consuming models such as LLM is still on with the proposal of novel processing units such as “Language Processing Units” (LPU) that are based on inference and promise to give more power than GPUs [207]. In line with these considerations, one can consider that the relative “delay” in the use of AI in DD, compared to the use of AI in the elucidation of protein structures, will probably be reduced in the coming years due to computer performance improvement and to the exponential involvement of AI in DD, leading to a strong development of corresponding databases. 

To summarise, soon, as an expected consequence of AI, it can be postulated that the roles of researchers in DD will likely evolve drastically once again. Researchers will be more and more involved in setting up potent and efficient learning processes (including database selection, ethical concerns, and AI model programming), and, of course, in the final criticism of AI outcomes.

## 7. Conclusions and Future Directions

The end of the 20th century may be considered a golden age for sciences such as genetics, biology, chemistry, and immunology. Taking advantage of their combination, the conventional approaches of DD led to indisputable worldwide improvements in quality of life and in life expectancy. Thus, some infectious diseases have been partly or totally eradicated, for example, smallpox, and new treatments against chronic and age-related pathologies such as cancers and Alzheimer are discovered regularly. However, despite these benefits, many pathologies remain incurable. Medicinal scientists now have to cross new frontiers to imagine original processes and in turn solve these concerns of public health.

AI has become an emerging topic of utmost interest in our daily lives as well as in medical sciences. AI roots are deep, but the continuous improvement of calculators and processors, the emergence of new calculation procedures and neuronal models, as well as its large popularisation within the scientific community, has rendered AI a promising tool for breaking the limits of the current drug discovery process. AI is already used to identify biological targets, design new therapeutic scaffolds, predict molecular toxicity, and help in organic syntheses. All these advances and uses have resulted in an exponential increase in scientific publications dealing with the use of AI in DD with some major outbreaks, among them, the discovery in 2024 of a new class of antibiotics, which is a rare event, never reported in the last 40 years. Thus, AI has also reached the capacity to identify new relevant targets and to design corresponding chemical agents able to counteract their activity. These successes pave the way towards many expected success stories for the treatment of several multifactor pathologies.

Over the years, AlphaFold has revolutionised the analysis of proteins thanks to the use of AI, taking advantage of a strong neuronal learning, based on the multiple proteins structures already resolved in the literature. The same will probably occur in the coming decade in DD, and the promising results in target and drug identification mediated by AI may suggest that we are at the beginning of a new era for medicine. A real scientific “Big Bang” is occurring, which breaks the rationale of the human thinking and consequently allows the emergence of unexpected therapies against multiple diseases.

## Data Availability

Not applicable, as no new data were reported here.

## Acronyms and Abbreviations

AMR	antimicrobial resistance
AI	Artificial Intelligence
ANN	Artificial Neural Network
CPPs	cell-penetrating peptides
CPU	Central Processing Unit
CADD	computer-aided drug discovery
cRNN	conditional RNN
CNN	Convolutional Neural Network
DL	Deep Learning
DARPA	Defense Advanced Research Project Agency
DC	drug candidate
DD	drug discovery
DTIs	drug–target interactions
GRU	Gated Recurrent Units
GAN	Generative Adversarial Net
GenAI	generative AI
GPU	Graphics Processing Unit
GCN	graph convolutional network
GNN	Graph Neural Network
HTS	high-throughput screening
hERG	human-ether-a-go-go-gene
IPF	idiopathic pulmonary fibrosis
LPU	Language Processing Unit
LLM	Large Language Models
LBDD	Ligand-Based DD
LSTM	Long Short-Term Memory
ML	Machine Learning
MPNN	Message Passing Neural Network
MRSA	methicillin-resistant *Staphylococcus aureus*
MD	molecular docking
NB	Naïve Bayes
NLP	Natural Language Processing
TNIK	NCK-interacting kinase
NMEs	New Molecule Entities
PM	pharmacophore modelling
POI	protein of interest
PPI	protein–protein interaction
PROTACs	PROteolysis TArgeting Chimeras
PMF	proton motive force
QSAR	Quantitative Structure-Based Relationship
RF	Random Forest
ROC	Receiver Operating Characteristic
RNN	Recurrent Neural Network
RL	Reinforcement Learning
SBDD	Structure-Based Drug Discovery
SBVS	Structure-Based Virtual Screening
SVM	Support Vector Machine
SD	synthetic data
VAE	Variational Autoencoder
VS	virtual screening
